# *PALLD* mutation in a European family conveys a stromal predisposition for familial pancreatic cancer

**DOI:** 10.1172/jci.insight.141532

**Published:** 2021-03-25

**Authors:** Lucia Liotta, Sebastian Lange, H. Carlo Maurer, Kenneth P. Olive, Rickmer Braren, Nicole Pfarr, Sebastian Burger, Alexander Muckenhuber, Moritz Jesinghaus, Katja Steiger, Wilko Weichert, Helmut Friess, Roland Schmid, Hana Algül, Philipp J. Jost, Juliane Ramser, Christine Fischer, Anne S. Quante, Maximilian Reichert, Michael Quante

**Affiliations:** 1 Klinik und Poliklinik für Innere Medizin II, Klinikum rechts der Isar, Technische Universität München, Munich, Germany.; 2 Division of Digestive and Liver Diseases, Department of Medicine, Vagelos College of Physicians and Surgeons, Columbia University, New York, New York, USA.; 3 Herbert Irving Comprehensive Cancer Center, Columbia University Irving Medical Center, New York, New York, USA.; 4 Institut für diagnostische und interventionelle Radiologie, Klinikum rechts der Isar, Technische Universität München, Munich, Germany.; 5 Institut für Pathologie und pathologische Anatomie, Technische Universität München, Munich, Germany.; 6 Deutschen Konsortium für Translationale Krebsforschung (DKTK), Partner site Munich, Technische Universität München, Munich, Germany.; 7 Chirurgische Klinik, Klinikum rechts der Isar, Technische Universität München, Munich, Germany.; 8 Innere Medizin III, Hämatologie und Onkologie, Technische Universität München, Munich, Germany.; 9 Klinik und Poliklinik für Frauenheilkunde, Klinikum rechts der Isar, Technische Universität München, Munich, Germany.; 10 Institut für Humangenetik, Ruprecht-Karls Universität, Heidelberg, Germany.; 11 Klinik für Innere Medizin II, Universität Freiburg, Germany.

**Keywords:** Gastroenterology, Oncology, Cancer, Molecular genetics

## Abstract

**BACKGROUND:**

Pancreatic cancer is one of the deadliest cancers, with low long-term survival rates. Despite recent advances in treatment, it is important to identify and screen high-risk individuals for cancer prevention. Familial pancreatic cancer (FPC) accounts for 4%–10% of pancreatic cancers. Several germline mutations are related to an increased risk and might offer screening and therapy options. In this study, we aimed to identity of a susceptibility gene in a family with FPC.

**METHODS:**

Whole exome sequencing and PCR confirmation was performed on the surgical specimen and peripheral blood of an index patient and her sister in a family with high incidence of pancreatic cancer, to identify somatic and germline mutations associated with familial pancreatic cancer. Compartment-specific gene expression data and immunohistochemistry were also queried.

**RESULTS:**

The identical germline mutation of the *PALLD* gene (NM_001166108.1:c.G154A:p.D52N) was detected in the index patient with pancreatic cancer and the tumor tissue of her sister. Whole genome sequencing showed similar somatic mutation patterns between the 2 sisters. Apart from the *PALLD* mutation, commonly mutated genes that characterize pancreatic ductal adenocarcinoma were found in both tumor samples. However, the 2 patients harbored different somatic *KRAS* mutations (G12D and G12V). Healthy siblings did not have the *PALLD* mutation, indicating a disease-specific impact. Compartment-specific gene expression data and IHC showed expression in cancer-associated fibroblasts (CAFs).

**CONCLUSION:**

We identified a germline mutation of the palladin (*PALLD*) gene in 2 siblings in Europe, affected by familial pancreatic cancer, with a significant overexpression in CAFs, suggesting that stromal palladin could play a role in the development, maintenance, and/or progression of pancreatic cancer.

**FUNDING:**

DFG SFB 1321

## Introduction

Pancreatic ductal adenocarcinoma (PDAC), the most common form of pancreatic cancer (1), still has a 5-year survival below 10% (2, 3) and represents the fourth leading cause of cancer-related deaths (4) in the European and US populations (5). Because of its rising incidence, epidemiologic studies calculated that it is going to be the second leading cause of death due to cancer by 2030 (6). The only curative option is surgical resection (7) combined with perioperative or adjuvant chemotherapy; however, most cases of pancreatic cancer (~80%) (8) are diagnosed at a locally advanced or unresectable stage (9).

Even though most cases of pancreatic cancers are sporadic with known risk factors (cigarette smoking, obesity, high meat intake, or low fruit and vegetable intake, as well as diabetes and chronic pancreatitis; ref. 10), up to 10% of all pancreatic cancers have an inherited genetic component (11, 12).

Familial pancreatic cancer (FPC) is clinically defined as 2 first-degree relatives with pancreatic cancer (13). While the genetic background responsible for most cases of FPC is still unknown, some of the genes responsible for its development have been identified. The most common familial syndromes are hereditary breast and ovarian cancers (*BRCA1* and *BRCA2* mutations) (14), Li-Fraumeni Syndrome (*TP53*), hereditary pancreatitis (*PRSS1*, *SPINK,* and — rarely — *CFTR* mutation) (15–18), Peutz–Jeghers syndrome (*STK11/LKB* mutation) (19, 20), hereditary nonpolyposis colorectal cancer (HNPCC) caused by germline mutations in DNA mismatch repair genes (*MSH2*, *MLH1*, *PMS1*, *PMS2* and *MSH6/GTBP*) (21, 22), ataxia telangiectasia (*ATM*), and familial atypical multiple mole melanoma syndrome (*p16/CDKN2A* mutation) (8, 23, 24). The individual risk of developing pancreatic cancer depends on the mutations’ level of penetrance, as well as on further environmental risk factors. Germline mutations associated with pancreatic cancer are *ATM* (2%–4%), *BRCA1* (0%–1%), *BRCA2* (8%–19%), *CHEK2* (2%–9%), and *PALB2* (3.1%–3.7%) (13). In addition to genetic factors, epigenetic or environmental factors may contribute to its development. The age of onset is typically a few years earlier than sporadic cases (FPC in patients 58–68 years old versus 61−74 years old) (25). Furthermore, European registries have observed an anticipation phenomenon. For instance, a large European study analyzed 106 FPC families through 3 generations and found that from one generation to the next, the age of death from PC was younger with each generation (26).

The discovery of familial pancreatic genes provides insights into the cellular pathways involved in the development of pancreatic cancer and is important in order to establish screening for patients who are genetically more susceptible and to offer genetic counseling for the family members. In 2001, the Pancreatic Cancer Genetic Epidemiology group (PACGENE) identified susceptibility genes in linkage studies (27) and succeeded in finding an association of 2 genetic loci with pancreatic cancer 7p21.1 (*HDAC9*) and 21q22.3 (*COL6A2*) (28). Further alterations such as *KDM6A* and *PREX2* were identified in whole-genome sequencing and copy number variation (CNV) analysis (29). Several segregation analyses suggest that more than 10% of patients with pancreatic cancer inherit the risk of pancreatic cancer in an autosomal dominant pattern (30). The main features are early age at onset (median age, 43 years) and further relatives affected (30). A recent case-control study analyzed 3030 patients suffering from pancreatic cancer. Germline mutations in 6 genes associated with pancreatic cancer (*ATM*, *BRCA1*, *BRCA2*, *CDKN2A*, *MLH1*, and *TP53*) were found in 5.5% of all patients with pancreatic cancer (11). However, an epidemiology and family study has demonstrated only a small increased risk of pancreatic cancer among first-degree relatives (total, 3355) of 426 patients with pancreatic cancer (standardized incidence ratio [SIR] of 1.88; 95% CI, 1.27–2.68) (31). The extension of sequencing studies led to the creation of a risk prediction tool (PancPRO), which assesses the risk of developing pancreatic cancer among individuals with family history of pancreatic cancer. Nevertheless, the ethical and moral implications on the healthy family members must be taken into consideration (32).

Here, we report on another genetic alteration, the susceptibility gene palladin (*PALLD*), which is a candidate gene for pancreatic cancer shown by significant linkage analysis and functional analysis (33, 34).

## Results

**Figure 1:**
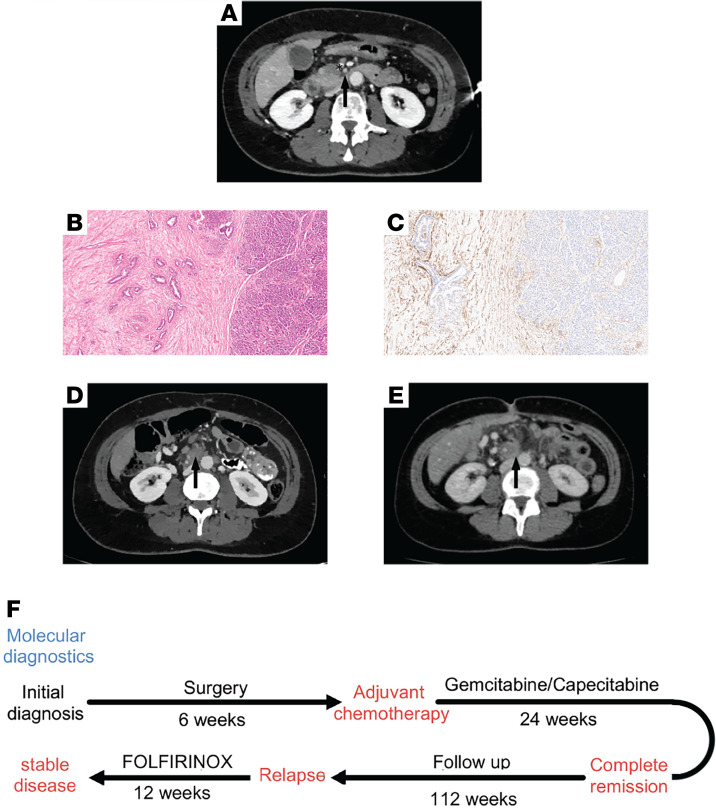
Clinical data of the patient. (**A**) Axial CT of the index patient prior to therapy, showing a hypodense mass in the head of the pancreas. Note deformity of the superior mesenteric vein (asterisk) indicating vascular wall infiltration. Hazy stranding (arrow), indicating desmoplasia, extends to the superior mesenteric artery. (**B**) Histopathological view of the surgical specimen of moderately differentiated (G2) pancreatic adenocarcinoma with H&E staining. Original magnification, 20×. There is a very strong induction of a fibroelastic desmoplastic stroma between the tumor and the adjacent inflammatory reaction. (**C**) IHC for PALLD in pancreatic tissue of the index patient with strong PALLD expression in CAFs, which is more enhanced than among the mesenchymal stroma cells of adjacent pancreatic tissue. Original magnification, 20×. (**D** and **E**) Axial CT scan showing a local relapse at the level of the primary tumor (**D**, arrow), which remains stable in the follow-up exam after 6 cycles of chemotherapy with FOLFIRINOX (**E**, arrow). (**F**) Timeline of index patient. Complete pancreatectomy was performed, followed by adjuvant chemotherapy with gemcitabine/capecitabine. Twenty-four months after surgery, the follow-up CT scan showed a local and lymphatic relapse; a systemic chemotherapy with FOLFIRINOX was suggested. The following CT staging showed a stable disease.

A 51-year-old woman presented to the hospital with a 1-week history of epigastric abdominal pain and acholic stools. She did not have unintentional weight loss, fatigue, or jaundice. Her medical history included hysterectomy and appendectomy, without a personal history of cancer. A CT scan of the abdomen and thorax revealed a mass in the uncinate process of the pancreas, radiographically consistent with pancreatic adenocarcinoma (Figure 1A). The mass encircled the superior mesenteric vein by approximately 90° with celiac, regional, and retroperitoneal lymph nodes. No distant metastases could be detected. The patient underwent an endoscopic ultrasound, which revealed a 3.5 cm diameter hypoechoic mass in the uncinate process. Fine-needle aspiration (FNA) biopsy at the time of endoscopic ultrasound was consistent with adenocarcinoma.

The consensus of an interdisciplinary tumor board was resection. Because of the familial history of pancreatic cancer, she underwent a complete pancreatectomy without any postoperative complications. The histological examination of the surgical specimen revealed a ductal adenocarcinoma (tumor size between 2–4 cm [pT2]), moderately differentiated (grade 2; G2). Of note, a very strong induction of a fibroelastic desmoplastic stroma between the tumor and the adjacent inflammatory reaction was observed (Figure 1B). Importantly, similar to previous reports defining cancer-associated fibroblasts (CAFs) (35–37), such fibroblasts expressed PALLD, which appeared to be more prominent than in the adjacent and distant normal pancreatic tissue (Figure 1C). Some of the resected lymph nodes were positive for malignancy (pN1 3/38), and there was evidence of residual tumor in the circumferential resection margin (CRM^+^) but less than 0.1 cm to residual tumor (R0). Following surgery, adjuvant chemotherapy with gemcitabine (1000 mg/m^2^) and capecitabine (830 mg/m^2^) for 6 cycles, according to the ESPAC-4 trial, was given (38).

A follow-up CT scan at 24 months after surgery showed a local and lymphatic relapse (Figure 1D). For this reason, a systemic chemotherapy with 6 cycles of FOLFIRINOX (irinotecan 180 mg/m^2^; oxaliplatin 85 mg/m^2^; folinic acid 400 mg/m^2^; 5-fluorouracil 400 mg/m^2^ bolus; and 5-fluorouracil 2400 mg/m^2^ over 46 hours) was recommended. Afterward, the patient underwent restaging with CT scans of the chest, abdomen, and pelvis, where the local relapse and the lymphatic metastases remained stable (Figure 1E). Since the patient did not tolerate the chemotherapy well, stereotactic radiotherapy was suggested. Currently, the patient is under stable conditions without further therapy within the yearly follow-up staging CT scans (Figure 1F).

### History of familial pancreatic cancer.

**Figure 2:**
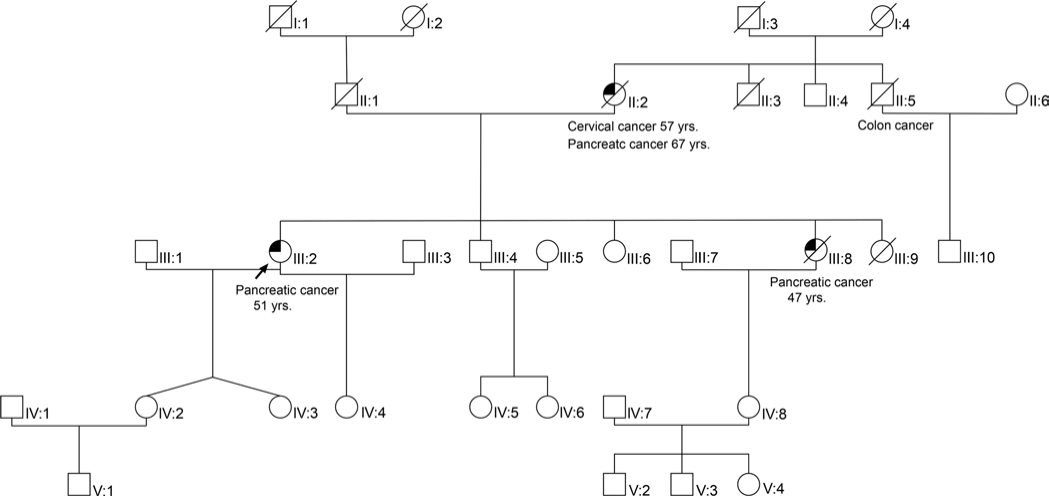
Genealogical tree of the index patient. Three members of this family were diagnosed with pancreatic cancer (II:2, III:2, III:8). Among them, 2 carried a *PALLD* mutation (III:2 germline and tumor tissue; III:8 tumor tissue). A germline mutation on II:2 and III:8 could not be evaluated since the patients died several years ago. Two healthy siblings of the index patient did not carry a germline *PALLD* mutation (III:4, III:6).

The patient’s family history included a sister and the mother who died from pancreatic cancer in their fifth and seventh decade of life, respectively (Figure 2). The sister (47 years) of the patient was diagnosed with pancreatic adenocarcinoma with peritoneal carcinosis. The diagnosis was confirmed through a laparoscopic biopsy of the peritoneum. The patient underwent 9 cycles of palliative chemotherapy based on gemcitabine regimen (1000 mg/m^2^), with an initial clinical response. Following a progression of the primary tumor and the peritoneal carcinosis, the chemotherapy was then switched to the OFF regimen (oxaliplatin 85 mg/m^2^; calcium folinate 200 mg/m^2^; 5-FU 2000 mg/m^2^). Due to further tumor progression with ascites, best supportive care was initiated.

The mother of the 2 patients was diagnosed with a pancreatic head tumor when she was 67 years old (Figure 2). Since the tumor was already locally advanced with vessel infiltration, the patient did not undergo any surgery. A few months later, the patient developed ascites, which was cytologically analyzed and compatible with the diagnosis of adenocarcinoma. Given that the sister and the mother of the index patient suffered from pancreatic cancer, and considering the patient’s young age at the time of the diagnosis, familial pancreatic cancer was suspected, and the patient’s tumor material was analyzed for genetic alterations. Considering the young age of the index patient and the familial history, we decided to perform somatic and germline whole exome sequencing through participation in the German Molecularly Aided Stratification for Tumor Eradication Research (MASTER) trial, which includes calling of potentially pathogenic germline mutations using specific bioinformatics pipelines and clinical evaluation by a medical geneticist.

### Molecular characterization of an inherited PALLD mutation.

Whole exome sequencing was performed on the surgical specimen and peripheral blood in order to identify mutations associated with FPC. Among several findings, a specific germline variant in the *PALLD* gene (NM_001166108.1:c.G154A:p.D52N) was detected. The *PALLD* gene itself has previously been described to be possibly associated with FPC, based on segregation data of another *PALLD* variant (c.C715T:p.P239S) (33).

Moreover, somatic mutations frequently found in PDAC, such as *KRAS* (C*G12D), *CDKN2A* (focal deletion 9p) and *SMAD4* loss, were also observed (Table 1, Table 2, Table 3, Table 4). Furthermore, potentially new candidate drivers of pancreatic carcinogenesis, like *RUNX1* and *ROBO2*, could also be found in the tumor (Table 1). Next, we analyzed the peritoneum specimen of the deceased sister by specific PCR and identified the identical *PALLD* mutation at the same position (c.G154A:p.D52N). In combination with previous significant linkage and functional analysis data suggesting that *PALLD* mutations cause FPC (33, 34), the *PALLD* gene was selected as the most likely cancer-causing alteration in this family. Indeed, assuming that the mutation is statistically independent of the development of pancreatic cancer in this family, the observed inheritance pattern has a probability of 0.0625, or 6.25%. Linkage analysis of this family under an autosomal dominant model of inheritance with full penetrance and without knowing which of the parent carried the mutation resulted in a LOD score of 0.9, with a corresponding *P* value of 0.04 rejecting the null hypothesis (the mutation is independent of the development of pancreatic cancer in the family) at a 0.05 significance level.

Having confirmed an inherited *PALLD* mutation, we further performed whole genome sequencing to better compare the sibling tumor sequences. The whole genome sequencing data show similar somatic mutation patterns between the 2 samples. Commonly mutated genes that characterize PDAC (*KRAS* and *CDKN2A*) could be found in both tumor samples (Table 1, Table 2, Table 3, Table 4). However, the 2 patients harbored different *KRAS* mutations (G12D in the index patient and G12V in the index patient’s sister) (39). In contrast, both harbor the exact same mutations of *COL6A2*, *ABO*, *CLPTM1L*, *BCAR1*, *FANCI*, and *ETAA1* gene, which we therefore defined as likely germline as well as a different mutation of *RREB1* (Table 1 and Table 2). However, the variants in *ABO*, *BCAR*, and *FANCI* were described as benign in ClinVar database analysis (https://www.ncbi.nlm.nih.gov/clinvar/), and the variants in *CLPTM1L* and *ETAA1* were not listed in ClinVar database (Table 1 and Table 2).

We were not able to examine the tumor of the mother, since no cytological sample was available anymore. Apart from the sister who suffered from pancreatic cancer, our patient also had 2 living siblings and 1 other sister, who died when he was 48 of unknown causes (Figure 2). Analysis for *PALLD* mutation in the healthy siblings — whose ages at the time of the screening were 58 and 61 years old — was negative, indicating that the identified *PALLD* mutation might have a disease-specific impact. Since we classified all other likely germline mutations that were found in both patients with PDAC as benign or could not identify a pathologic variant in our ClinVar database search, we refrained from analyzing those in the healthy siblings. In the absence of clearly beneficial treatments or effective preventive strategies, genetic testing of the additional family members was not justified.

### PALLD expression in PDAC specimens.

Previous studies have shown that palladin is highly expressed in the CAFs of pancreatic tumors and other invasive tumor types, like renal cancer (36). Its upregulation is associated with more invasive PDACs rather than the less aggressive ones (40), suggesting that palladin overexpression may have a role in CAF-mediated tumor invasiveness. Since PDAC is characterized by a strong desmoplastic reaction that creates a dense microenvironment and epithelial-mesenchymal transition (EMT) (41), the *PALLD* mutation might promote tumor-stromal interactions, leading to tumor invasiveness and metastasis.

**Figure 3:**
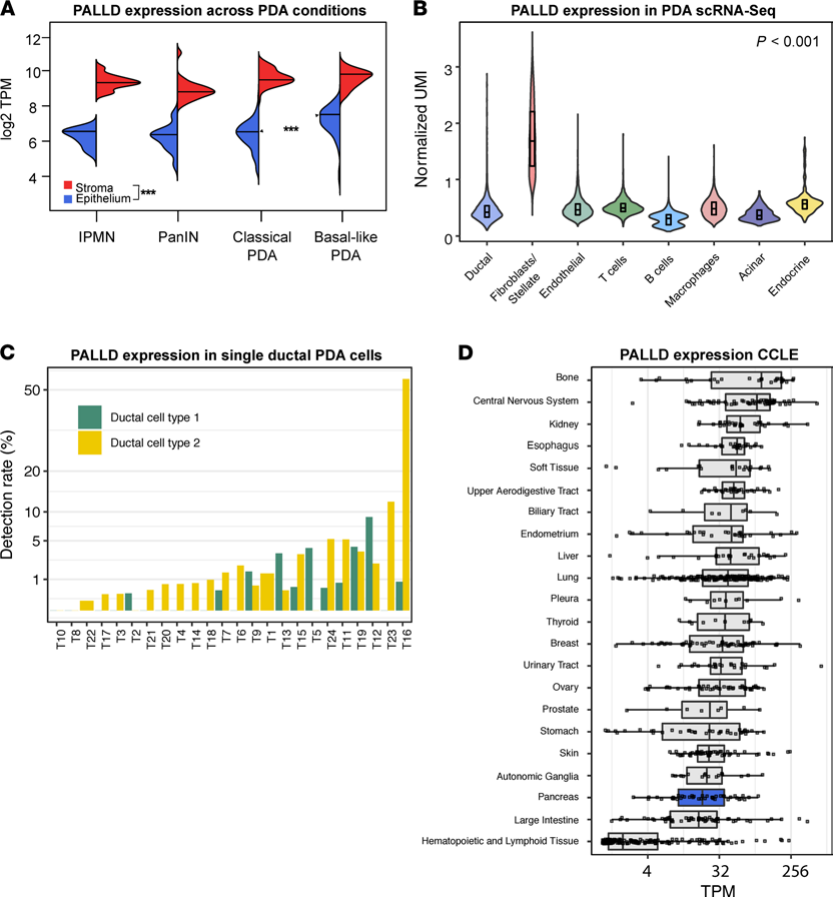
PALLD expression. (**A**) Human PALLD expression in transcripts per kilobase million (TPM, log_2_ scale) in epithelial and stromal samples gathered from pancreatic intraepithelial neoplasia (PanIN), intraductal papillary mucinous neoplasia (IPMN), and PDAC using laser capture microdissection and subsequent RNA sequencing. ***P < 0.001 from pairwise Wilcoxon rank-sum test with Bonferroni correction. (**B**) Human PALLD expression in unique molecular identifiers (UMI, log_2_ scale) in the indicated cell types from single-cell RNA-Seq data derived from human PDA specimen. *P* < 0.001 using Kruskal-Wallis rank-sum test. (**C**) Human PALLD detection rates among ductal cells from PDA specimen. Ductal cell type 1 represents normal ductal cells, while ductal cell type 2 comprises pre-malignant and malignant ductal cells (44). Detection rates describe the fraction of cells in which PALLD is expressed at levels above the fifth percentile of PALLD expression observed in cancer-associated fibroblasts (CAF). (**D**) Human PALLD expression in TPM in cancer cell lines from various tissues as profiled by the Cancer Cell Line Encyclopedia (CCLE).

In order to gain further insight into potential consequences of our observed *PALLD* mutation, we turned to compartment-specific gene expression data from human precursor lesions and PDAC specimen, which was generated using laser-capture microdissection (LCM) and subsequent RNA sequencing (RNA-Seq), as described previously (42, 43). Consistent with previous reports, we observed a strong preference of expression for the stromal compartment across all conditions (Figure 3A) without significant upregulation during progression from precursor to PDAC stroma. A similar pattern could be observed for epithelial cells, with the notable exception that basal-like tumors expressed significantly higher levels of PALLD when compared with classical tumors.

For an even higher resolution of cell type–specific PALLD expression in PDAC, we examined single-cell RNA-Seq data from 24 human PDAC specimens as described by Peng et al. (44) and again found CAFs to express PALLD at the highest level (Figure 3B). However, substantial PALLD expression can occur in ductal cells. Using the fifth percentile of PALLD expression in CAF as a rigorous cutoff, PALLD could be detected among malignant — and more rarely — normal ductal cells in many of the 24 PDA specimen (Figure 3C). In PDA specimen T16 for example, about 50% of tumor cells expressed PALLD at levels comparable with CAF. Among cancer cell lines, a wide array of tissues giving rise to carcinomas did show robust PALLD expression, with a major gap only appreciated between hematological malignancies and all others (Figure 3D).

Taken together, these findings corroborate the PDAC CAFs in the stroma as the predominant source of PALLD expression in PDAC specimen. However, PALLD expression does occur in malignant ductal cells at high levels, which correlate with a dedifferentiated, more aggressive class of tumors, consistent with cell-autonomous, tumor-promoting qualities of *PALLD*.

## Discussion

We identified a family with pancreatic cancer with a germline mutation of *PALLD*, located in a pancreatic cancer susceptibility locus at 4q32-34, in 2 sibling patients suffering from pancreatic cancer, whereas 2 healthy siblings were not carrying the mutation. A germline mutation in the palladin gene has already been described once among members of FPC and not in the unaffected members (33, 34). Both cases report on a missense mutation in exon 2 but localized on different positions (c.C715T:p.P239S and c.G154A:p.D52N). Nonetheless, a lack of palladin somatic mutations in FPC individuals (45, 46) and previous linkage studies (40) have been used to argue that the Family X mutation in palladin cannot be a driver of PDAC. In line with such reports and our finding of a substantial overexpression of PALLD in the tumor stroma (CAFs) in compartment-specific gene expression data from human precursor lesions and PDAC specimen, and in the tissue of the affected patient, we propose here that a palladin germline mutation, just like the one in Family X, could convey a stromal predisposition to developing pancreatic cancer.

Palladin is a fundamental protein of the cell cytoskeleton that is required for organizing the actin cytoskeleton. It is involved in the regulation of cell shape, adhesion, and contraction (47). It binds to α-actinin, ezrin, and other cytoskeletal proteins in order to form the actin filaments necessary for the cell form and movement (47). Pogue-Geile et al. (33) performed functional tests with cells expressing the P239S mutant palladin protein and found that the binding site for α-actinin was affected. The mutated protein showed an increased motility and alterations in cell adhesion, suggesting that it could have a role in the carcinogenesis as a proto-oncogene. Indeed, other studies indicate a role for palladin in tumor invasiveness through its overexpression in the stroma, explaining why its higher expression is associated with a poorer prognosis (35). This functional and expression study showed that a specific palladin isoform (isoform 4) is dramatically upregulated in cancer-associated fibroblasts in the early stages of pancreatic cancer (35, 37). In addition, it was demonstrated that the palladin protein was strongly expressed in stromal cells in most cases of pancreatic cancer (96.6% of the 177 evaluated pancreatic cancers) (46). In our compartment-specific gene expression data from precursor lesions and PDAC specimen, PALLD expression was mostly upregulated in CAFs, supporting the concept that PALLD indeed promotes tumor–stromal interactions, leading to tumor invasiveness and metastasis. This also correlated with a strong PALLD expression in CAF in the tissue of the index patient compared with normal pancreatic tissue.

Similar to our patients, the vast majority of PDAC harbor a *KRAS* mutation, which was also found in the Family X of the study of Pogue-Geile et al. (33), suggesting that we likely observed the well-known *KRAS*-driven pancreatic carcinogenesis. Exome sequencing results in this study point to an interesting relationship of *KRAS* mutations with palladin mutation, as both tumors within the family harbor the exact same mutations of *COL6A2*, *ABO*, *CLPTM1L*, *BCAR1*, *FANCI*, and *ETAA1* genes, which have previously been described in PDAC. Interestingly, the *KRAS* mutation in both tumors were distinct, suggesting that *KRAS* is needed for tumor development but is independent and likely secondary to *PALLD* alterations. Previous studies have shown increased PALLD expression in CAFs of pancreatic tumors and other invasive tumor types (40), suggesting a role in tumor aggressiveness and invasiveness. Indeed, we detected a much higher PALLD expression among CAFs of 24 human PDA specimens. However, malignant ductal cells could also express PALLD at high levels, suggesting that its expression is associated with a more dedifferentiated — and, thus, more aggressive — tumor. Further studies are needed in order to investigate the interaction of stromal palladin with the known pancreatic cancer pathways. Nevertheless, this study provides evidence that *PALLD* is a pancreatic cancer susceptibility gene, likely through the presence of an abnormal palladin gene in stromal CAFs, therefore defining a carcinogenic tumor microenvironment that favors distinct mutagenic alteration in pancreatic ductal cells.

## Methods

### Whole exome sequencing.

Somatic and germline whole exome sequencing data were generated through participation in the MASTER trial, led by the German Cancer Research Center and the German Cancer Consortium in Heidelberg, Germany. The detailed workflow has been described earlier (48). Briefly, following a standard protocol, unfixed tissue was flash frozen and submitted to a central sample processing laboratory for DNA isolation. Whole exome libraries were prepared from tumor DNA and from peripheral blood mononuclear cells using the Agilent SureSelect Human All Exon V6 library preparation kit. The resulting libraries were sequenced an Illumina NovaSeq 6000 Sequencer (2 × 100 paired-end) to a coverage of approximately 150 (tumor) and approximately 100 (normal) depth of coverage, respectively.

Tumor DNA for the sister of the index patient was isolated from archival FFPE blocks: 2 μm sections were prepared with a rotary and subjected to histological and IHC analysis. H&E staining was performed on deparaffinized sections according to standard protocols. Eight 10 μm sections of FFPE tumor specimens were deparaffinized and digested with Proteinase K (QIAGEN) overnight. DNA isolation was performed using the Maxwell 16 RSC extraction system (Promega).

### Mutation analysis.

Whole genome libraries were prepared using the Illumina TruSeq Nano DNA kit to manufacturer instructions, and we sequenced 2 lanes of a Illumina HiSeq X Ten sequencer, resulting in a whole genome coverage of approximately 60. For both the index patient and the sister, the same bioinformatic workflow was used: The GATK Best Practices suggestions were followed for mutation calling. After read trimming using Trimmomatic 0.38 (LEADING:25, TRAILING:25, MINLEN:50), BWA-MEM 0.7.17 was used to map reads to the reference genome (GRCh38.p12). Picard 2.18.26 and GATK 4.1.0.0 were used for postprocessing (CleanSam, MarkDuplicates, BaseRecalibrator) using default settings. Somatic mutations were called using MuTect2 v4.1.0.0. Mutations with at least 2 reads supporting the alternate allele and an overall base coverage of at least 10 in the tumor, and where available in the germline sample, were required. Putative germline variants were evaluated using both the gnomAD database (49) using a cutoff of 1% population frequency and germline information from the index patient. SNVs and Indels ≤ 10 bp were annotated using SnpEff 4.3t, based on ENSEMBL 92. Copywriter 2.6.1.2 was used for the detection of CNVs.

### Histopathology.

After deparaffinization and rehydrating to water, FFPE tissue sections (2 μm) from the index patient were heated by microwaving for 30 minutes in pH 6 target retrieval buffer (Agilent DAKO) to unmask antibody epitopes. Nonspecific binding was blocked by protein blocking solution (5% v/v rabbit serum/antibody diluent; REAL antibody diluent, Agilent DAKO). Sections were then washed in PBS (MilliporeSigma) after each step. For PALLD IHC, sections were deparaffinized and stained with the rabbit anti-palladin (1:200 dilution, Cell Signaling Technology, 8518) antibody using a Leica Bond-RXm. The histological sections were scanned with Leica-AT2 Slidescanner, and the images were exported with Leica ImageScope Software Version 12.4.0.7018.

LCM and subsequent RNA-Seq from human pancreatic resections was performed as described previously (42). Briefly, cryosections of OCT-embedded tissue blocks from pancreatic resections collected at the Columbia Pancreas Center were transferred to PEN membrane glass slides and stained with cresyl violet acetate. LCM was performed on a PALM MicroBeam microscope (Zeiss), collecting at least 1000 cells per compartment. RNA was extracted and libraries were prepared using the Ovation RNA-Seq System V2 kit (NuGEN). Libraries were sequenced to a depth of 30 million, 100 bp, single-end reads.

### Single-cell RNA-Seq.

Raw UMI counts per gene, along with sample and cluster annotations of 24 human PDAC and 11 human normal pancreas samples from the study by Peng et al. (44), were downloaded from the Chinese National Genomics Data Center (Genome Sequence Archive, accession no. CRA001160). Raw counts underwent denoising using the provided cluster annotation and the *DCA* Python (50) software and subsequent normalization using the *scran* R (51) package.

### Public cell line expression data.

Processed expression data from the Cancer Cell Line Encyclopedia together with sample annotation were retrieved from the The Cancer Dependency Map Project website (version Public 20Q3; ref. 52).

### Statistics.

PALLD expression was compared between various conditions. For pairwise comparisons, log_2_ TPM and UMI were compared using nonparametric tests as implemented in the R stats package; for pairwise comparisons between compartments, Wilcoxon rank-sum tests were used with subsequent correction for testing multiple hypotheses by the Bonferroni method. For global comparisons, a Kruskal-Wallis rank-sum test was used.

### Study approval.

Patients in the study provided written informed consent for research use of personal data and biomaterial, and the study was approved by the ethical committee of the Klinikum rechts der Isar, Technical University of Munich.

## Author contributions

LL wrote the manuscript and collected data. RS, HA, PJJ, MR, and MQ performed study design, edited the manuscript, and guided patient treatment. HF performed surgery. SL and NP performed and evaluated sequencing analysis. AM and WW evaluated histopathology. RB performed and evaluated imaging. JR and ASQ performed and evaluated genetic analysis. HCM and KPO performed compartment-specific gene expression. CF performed epidemiological evaluation. MJ and KS evaluated histopathology.

## Supplementary Material

Trial reporting checklists

ICMJE disclosure forms

## Figures and Tables

**Table 1:**
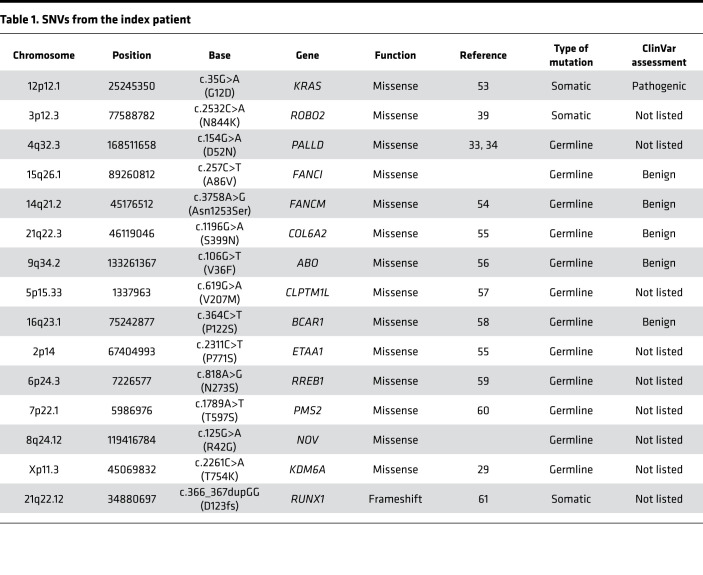
SNVs from the index patient

**Table 2:**
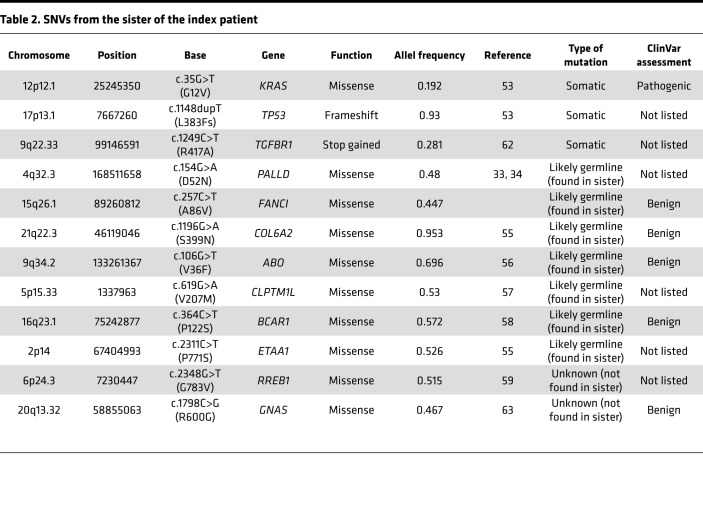
SNVs from the sister of the index patient

**Table 3:**
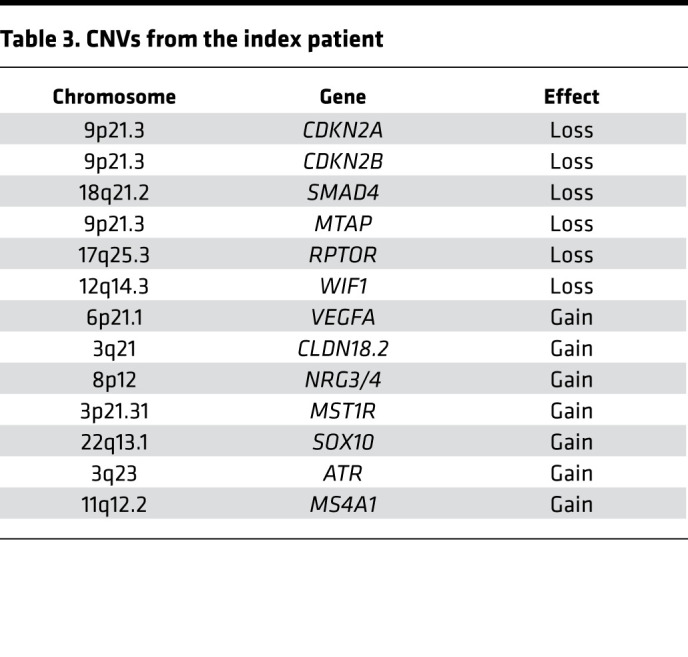
CNVs from the index patient

**Table 4:**
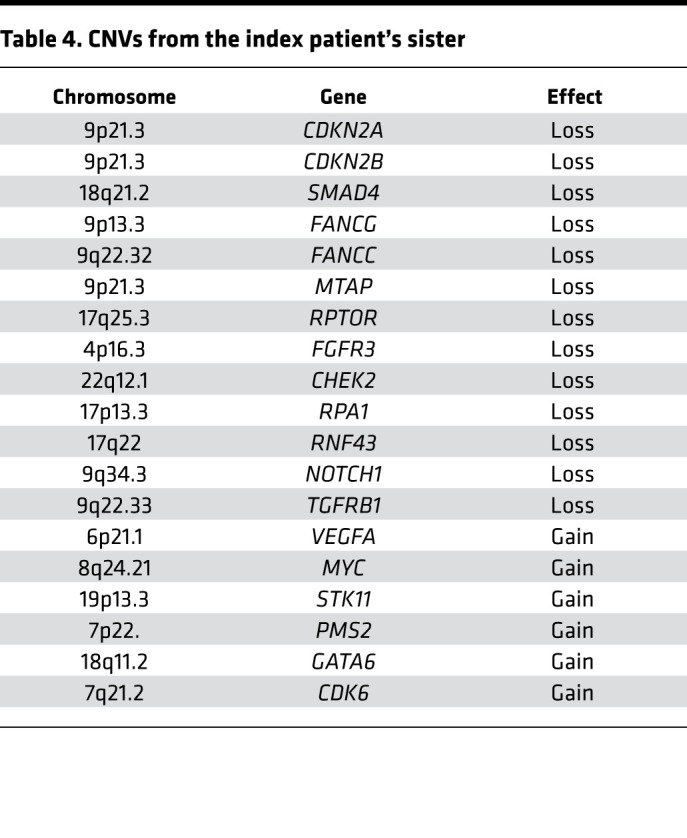
CNVs from the index patient’s sister

## Version 1 03/25/2021

In-Press Preview

## Version 2 04/22/2021

Electronic publication
